# Molecular Mechanisms Underlying the Link between Diet and DNA Methylation

**DOI:** 10.3390/ijms19124055

**Published:** 2018-12-14

**Authors:** Fatma Zehra Kadayifci, Shasha Zheng, Yuan-Xiang Pan

**Affiliations:** 1 Department of Public Health Sciences, California Baptist University, Riverside, CA 92504, USA; fzk@illinois.edu (F.Z.K.); szheng@calbaptist.edu (S.Z.); 2 Department of Food Science and Human Nutrition, University of Illinois at Urbana-Champaign, IL 61801, USA; 3 Division of Nutritional Sciences, University of Illinois at Urbana-Champaign, IL 61801, USA; 4 Illinois Informatics Institute, University of Illinois at Urbana-Champaign, IL 61801, USA

**Keywords:** epigenetics, gene expression, nutrition, transcription, disorders, mechanisms

## Abstract

DNA methylation is a vital modification process in the control of genetic information, which contributes to the epigenetics by regulating gene expression without changing the DNA sequence. Abnormal DNA methylation—both hypomethylation and hypermethylation—has been associated with improper gene expression, leading to several disorders. Two types of risk factors can alter the epigenetic regulation of methylation pathways: genetic factors and modifiable factors. Nutrition is one of the strongest modifiable factors, which plays a direct role in DNA methylation pathways. Large numbers of studies have investigated the effects of nutrition on DNA methylation pathways, but relatively few have focused on the biochemical mechanisms. Understanding the biological mechanisms is essential for clarifying how nutrients function in epigenetics. It is believed that nutrition affects the epigenetic regulations of DNA methylation in several possible epigenetic pathways: mainly, by altering the substrates and cofactors that are necessary for proper DNA methylation; additionally, by changing the activity of enzymes regulating the one-carbon cycle; and, lastly, through there being an epigenetic role in several possible mechanisms related to DNA demethylation activity. The aim of this article is to review the potential underlying biochemical mechanisms that are related to diet modifications in DNA methylation and demethylation.

## 1. Introduction

It has been well known that cytosine (C) in the genome, as part of the genetic code, also transfers epigenetic information through the chemical modification of its pyrimidine ring [1,2]. Methylation of the fifth position of cytosine (5mC) is a highly conserved epigenetic modification of DNA that is found in most prokaryotic and eukaryotic models [3], and it has a pivotal impact on genome stability, gene expression, and development [1]. Methylation of the DNA takes place almost completely in the symmetric cytidine–guanine dinucleotide (CpG) context, and is assessed to occur at nearly 70–80% of CpG sites throughout the genome [4]. Additionally, on bacterial and plant DNA, methylation can also occur at an adenine site, which regulates different bacterial and plant DNA functions. Recently, it has been discussed that there is indirect evidence suggesting the presence of adenine site methylation on mammalian DNA. However, the functionality of this base remains unclear on mammals [5]. 

DNA methylation is a crucial element in the control of the precise expression of genetic information, and both hypermethylation and hypomethylation have been associated with improper gene expression [6]. Irregular changes in genetic methylation patterns or an unusual analysis of DNA methylation signals are associated with many disorders and cancers [7]. Furthermore, the regulation of DNA methylation, crucially, is associated with other metabolic pathways, such as the one-carbon cycle, which have a significant impact on epigenetic regulations [8]. Two types of risk factors can alter the epigenetic regulation of methylation pathways. The first factor is the genetic factors, such as polymorphism and genetic mutations, which can cause aberrant DNA methylation [7,8]. Secondly, there are potentially modifiable factors, such as the modification of essential nutrients that are involved in the metabolism of methyl groups [9].

Nutrition is a strong player not only for its influence on gene expression, but more importantly, because early nutrition alterations could be responsible for the later development of chronic diseases through epigenetic mechanisms [10]. Both animal and human studies have investigated the effects of nutrition on DNA methylation pathways, but to our knowledge, relatively few have focused on the biochemical mechanisms. Understanding the biological mechanisms is important for future studies to clarify how nutrients function in epigenetics. Thus, the aim of this article is to review the underlying biochemical mechanisms of diet-related modifications in DNA methylation and demethylation. We also aim to go through all of the possible nutrient and DNA methylation interactions in more detail, and examine the underlying mechanisms of these relations by including both recent human and animal studies. Additionally, we opt to clarify the effect of diet on the DNA demethylation pathway, which has not been cleared in previous review articles. 

## 2. Mechanisms of DNA Methylation

### 2.1. What is DNA Methylation?

DNA methylation is a biological process that occurs in the addition of methyl groups to DNA. Methylation marks on DNA occur mainly on the 5′ position of cytosine residues of a CpG. It contributes to the epigenetics by regulating the gene expression without changing the DNA sequence [1]. In prokaryotes, DNA methylation is essential for transcription, the direction of post-replicative mismatch repair, the regulation of DNA replication, cell-cycle control, bacterial virulence, and differentiating self and non-self DNA [2]. In mammalians, DNA methylation is crucial in many key physiological processes, including the inactivation of the X-chromosome, imprinting, and the silencing of germline-specific genes and repetitive elements [1]. Besides, DNA methylation has been found to be present in actively transcribed gene bodies, and it may play a part in suppressing cryptic transcriptional initiation from the interior of genes [3]. DNA methyltransferase (DNMT) enzymes, which are pivotal for normal development, catalyze the transfer of the methyl group to DNA [4]. Importantly, DNMT’s interaction with other components and modifications are required to maintain DNA methylation [6].

The methylation cycle starts with the transportation of a methyl group by tetrahydrofolate, which carries it on its N-5 atom. Since the transfer potential of tetrahydrofolate is not sufficiently high for most biosynthetic methylations, *S*-adenosyl-l-methionine (SAM) supplies the main activated methyl donors for DNA methylation, which is synthesized by the transfer group from ATP to the sulfur atom of methionine. The positively charged sulfur atoms and the methyl groups become more electrophilic, and thus, the high transfer potential of the *S*-methyl group enables it to be transferred to a wide variety of acceptors. After SAM transfers the methyl group to an acceptor, *S*-adenosylhomocysteine (SAH) forms, which then hydrolyzes to homocysteine and adenosine [7].

Methionine can be renewed by the transfer of a methyl group to homocysteine from *N*^5^-methyltetrahydrofolate [7]. Additionally, this reaction is catalyzed by methionine synthase (MS) and requires vitamin B12 as a cofactor in animals. However, the same system in plants is cobalamin-free [8]. In mammalians, not only vitamin B12 has an important cofactor role: vitamin B2, which is a cofactor of methylenetetrahydrofolate reductase (MTHFR), and vitamin B6, which is a cofactor of serine hydroxymethyltransferase (SHMT), also have crucial roles as precursors of SAM [9].

**Figure 1:**
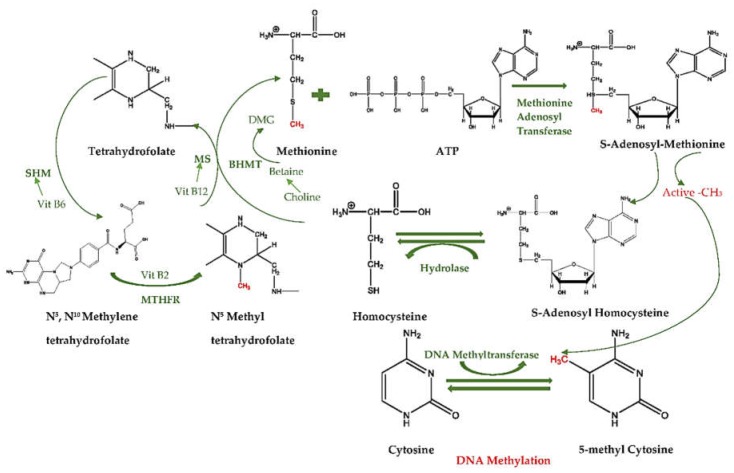
One-Carbon Cycle and DNA Methylation. MTHFR, methylenetetrahydrofolate reductase; SHMT, serine hydroxymethyltransferase; MS, methionine synthase; BHMT, betaine homocysteine methyltransferase; DMG, dimethylglycine.

Betaine is also an important methyl donor mediated by betaine homocysteine methyltransferase (BHMT), which is an alternative pathway that supplies the transfer of homocysteine to methionine [10]. Betaine can be produced through the irrecoverable oxidation of choline, and converts into dimethylglycine (DMG) after it provides a methyl group to homocysteine [11]. Therefore, any changes in these cofactors or enzymes may change the activity of folate and the methionine cycle, and thus further DNA methylation (Figure 1).

As it has been revealed, to methylate CpG sites in DNA, methyl groups need to enter the methionine cycle in the conversion of homocysteine into methionine; here, they are made highly reactive by the addition of adenosyl groups, which are specific bases in DNA that are modified by SAM. Furthermore, the CpG base to be methylated is flipped out of the DNA double helix into the active site, where it can accept the methyl group from SAM [7]. Primarily, DNA methyltransferases catalyze the transference of the methyl groups from SAM to methylate cytosine in DNA [2].

### 2.2. Why Are DNA Methyltransferases Essential for DNA Methylation?

DNMTs are the enzymes that maintain the formation of DNA methylation [12], and have key roles in transcriptional silencing, transcriptional activation, and post-transcriptional gene regulation [13]. Mammalians encode five DNMTs: DNMT1, DNMT2, DNMT3A–DNMT3B (de novo methyltransferases), and DNMTL. DNMT1, DNMT3A, and DNMT3B are the three active enzymes that maintain DNA methylation. DNMT3L has no catalytic activity and functions as a regulator of DNMT3A and DNMT3B, whereas DNMT2 acts as a tRNA transferase rather than a DNA methyltransferase [14]. The coordination of all of the DNMT enzymes is crucial for the regulation of DNA methylation patterns [15]. Although both DNMT1 and DNMT3 enzymes have different and non-redundant functions, they act cooperatively in some respects, such as both enzymes being required for the maintenance of the global hypomethylation patterns in mouse embryonic stem cells [16]. However, the functioning of both enzymes together and the mechanisms that maintain methylation patterns are still debatable [17].

Furthermore, in the past few years, studies have increased their attention on assessing the functional role of DNMTs by combining molecular approaches with a broad analysis of methylation patterns [13] such as looking through the pathways through which DNMTs catalyze the transference of the methyl groups to DNA from SAM, which is required as a cofactor [2]. The research has revealed that DNMTs are mechanistically multi-directional, which supports the notion that these enzymes have a significant role in epigenetic regulations [13].

### 2.3. How DNMT1 Functions in DNA Methylation

DNMT1 consists of a C-terminal methyltransferase domain and an N-terminal regulatory domain that mediates interactions with proteins, substrates, DNA, etc. [18]. DNMT1 is mainly responsible for replicating pre-existence methylation patterns, from hemimethylated CpG sites to the newly synthesized strands [19]. Additionally, DNMT1 contains functional subdomains that mediate molecular interactions. In order to have a deeper understanding of metabolic pathways, it is crucial to understand the role of these subdomains, which consist of the DNMT1-associated protein 1 (DMAP1) binding domain, replication foci targeting sequence (RFTS) domain, CXXC domain, Bromo-adjacent homology (BAH) domain, and catalytic domain [13]. To give a brief overview of their molecular roles: the DMAP1 binding domain is a protein that links DNMT1 to histone acetylation [20]. The RFTS domain targets the DNMT1 to replication foci, and thus promotes post-replicative maintenance methylation [21]. The CXXC domain is a zinc-finger domain, which mediates binding to unmethylated CpG dinucleotides [22]. Unfortunately, the functions of the BAH domain are still unknown.

Furthermore, the DNMT1 activity can be regulated by other molecular interactions. For instance, the DNMT1-interacting protein E3 ubiquitin-protein ligase (UHRF1), which is essential for methylation, flips the methylated base out of the DNA helix, and thus targets DNMT1 to its physiological substrate. Moreover, both UHRF1 depletion and overexpression indicated a global loss of DNA methylation [23,24], which indicates the importance of the interaction of DNMT1 with proteins in DNA methylation pathways. However, a small number of studies have shown the biochemical mechanisms leading to aberrant DNA methylation when DNMT1’s expression is reduced. To illustrate, an epigenetic study has demonstrated that mice with low DNMT1 expression at 10% of the wild-type level established a marked reduction in genome-wide DNA methylation, and revealed a significant increase in genomic instability and the activation of proto-oncogenes [25]. On the other hand, the study did not show whether DNMT reduction causing abnormal DNA methylation was because of the repression of sub-binding domains, the catalytic site, or DNMT-interacting proteins. It is necessary to know the underlying reasons in order to be able to understand the functioning of DNA methylation.

### 2.4. How DNMT3 Functions in DNA Methylation

DNMT3A and DNMT3B, de novo methyltransferases, are responsible for the methylation of unmodified DNA and the establishment of DNA methylation patterns [19]. These enzymes are mainly essential for de novo methylation, but several studies have shown that DNMT3 enzymes are also crucial for the stable inheritance and active remodeling of DNA methylation patterns in differentiated cells [26,27]. Structurally, both enzymes have a C-terminal catalytic domain that is similar to DNMT1, and a variable region at the N-terminus [28]. Additionally, two subdomains have also been described for DNMT3 enzymes that are important for chromatin interactions, which are the Pro-Trp-Trp-Pro (PWWP) and ATRX-DNMT3-DNMT3L (ADD) domains [29].

The targeted impairment or inactivation of both DNMT3A and DNMT3B in mammal embryonic stem cells blocks de novo methylation [30] and leads to the gradual loss of DNA methylation [14]. It has been indicated that the impaired activity of DNMT3A is a causal factor of tumorigenesis that causes global hypomethylation in specific types of cancer [15,31]. The deletion and overexpression of DNMT3B have been shown, respectively, to suppress and stimulate a specific type of cancer [32,33]. Knockout studies in mice have shown that de novo DNA methylation is pivotal for development, while DNMT3A-deficient mice die several weeks after birth, and DNMT3B-deficient mice die in utero [34]. To sum up, it is assumed that altering the regulation of DNMT3 enzymes may affect DNA methylation activity that results in several diseases, but further studies are needed to clarify the mechanisms between the activity of DNMT3 enzymes and DNA methylation.

### 2.5. What Is DNA Demethylation?

DNA demethylation is the process of removal of the methyl group. Currently, the DNA demethylation process is not clearly identified because of the multiple different pathways that contribute and act redundantly during this process [35]. DNA methylation has always been an active chemical process, which was originally regarded as an irreversible modification [35], but now it is found that DNA demethylation can occur, which follows either a passive or active process [36].

Mainly, DNA demethylation is passively diluted after DNA replication. However, recently, it has been revealed that DNA demethylation may also occur through the active process [14]. Unfortunately, studies about DNA demethylation mechanisms are conflicting, and its interaction with modifiable factors in mammals is still not well understood [37].

### 2.6. How Active DNA Demethylation Occurs

**Figure 2:**
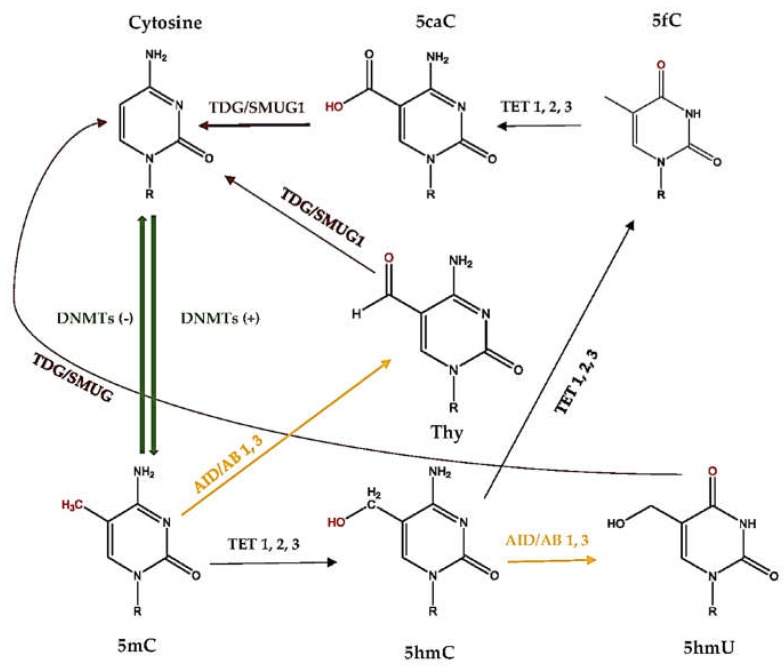
Active DNA demethylation process. 5mC, 5-methylcytosine; 5hmC, 5-hydroxymethylcytosine; 5hmU, 5-hydroxymethyluracil; 5fC, 5-formylcytosine; 5caC, 5-carboxylcytosine; Thy; thymine; DNMT, DNA methyltransferase; TET, ten-eleven translocation; AID, activation-induced deaminase; TDG, thymine DNA glycosylase; SMUG1, single-strand selective monofunctional uracil DNA glycosylase.

It has been proposed that the direct conversion of 5-methylcytosine (5mC) to cytosine does not occur [35]. Instead, active demethylation follows a series of chemical reactions that further transform 5mC to 5-hydroxymethylcytosine (5hmC), 5-hydroxymethyluracil (5hmU), 5-formylcytosine (5fC), 5-carboxylcytosine (5caC), and thymine (Thy), by deamination and/or oxidation reactions. Additionally, ten-eleven translocation (TET) and activation-induced deaminase (AID) enzymes catalyze these reactions. Later, these products are believed to recognized, mainly, by the base excision repair (BER) pathway to replace the modified base with naked cytosine [38]. Similarly, uracil misincorporation is repaired by BER, involving a series of enzymatic steps [39] (Figure 2). However, reserving the reactions in global DNA demethylation has generated conflicting results [40].

Active DNA demethylation is often carried out by members of the ten-eleven translocation (TET) family of enzymes, which functions against the actions of the DNMTs and prevents genome hypermethylation. The three members (TET1, TET2, and TET3) of the TET family oxidize 5mC to promote DNA demethylation [41]. The interaction between TET and demethylation has been shown, as TETs oxidize 5mC and significantly reduce the level of 5mC, which may result in hypomethylation. On the other hand, a loss of TETs may result in hypermethylation [42]. Furthermore, the balance between TET and other demethylation enzymes is also important. For instance, DNMTs and TETs are necessary to define the methylation landscape of gene regulatory regions. The examinations of mice that lack both DNMT3A and TET have suggested that these enzymes act in both a counteractive and synergetic way [43]. Moreover, it has been proposed that the conversion of 5mC to 5hmC by TET1 initiated an oxidative deamination process mediated by the coordinated action of activation-induced deaminase (AID)/APOBEC proteins and the BER pathway, which led to DNA demethylation in the adult brain [44]. AID deaminates cytosine to uracil and, to a smaller extent, 5mC to Thy, by simple hydrolysis [35]. The role of AID in demethylation and expression in embryonic stem cells is controversial. Popp et al. demonstrated that the absence of AID has increased DNA methylation levels, mainly in introns and repetitive elements and also in exons, but not in the promoter regions [45]. However, in vitro findings by Nabel et al., which also apply in vivo, showed that the role of AID in the direct demethylation of 5mC and 5hmC may be limited [46].

Evidence suggests that some of the products of 5mC oxidation could essentially revert back to cytosine [35]. It is known that thiol reagents and DNMT3A/DNMT3B can convert 5hmC (with the loss of formaldehyde) and 5caC (with the loss of CO_2_) to cytosine in the absence of SAM [47]. Moreover, uracil generated from cytosine can be excised by thymine DNA glycosylase (TDG) and single-strand selective monofunctional uracil DNA glycosylase (SMUG1). The TDG enzyme is one of the main BER glycosylases involved in the base excision step, which is able to revert 5caC, 5fC, and Thy back to cytosine; it also plays a crucial role in cellular defense against genetic mutation caused by self-induced deamination of 5mC and cytosine [48]. In addition to BER, nucleotide excision repair (NER), Gadd45a, and non-canonical mismatch repair (ncMMR) systems are suggested to have roles in the reverse step of active DNA demethylation [35]. However, both the BER and alternative repair pathways are not well understood, and it is not clear how modified factors may affect the regulation of these pathways.

## 3. What Are the Underlying Mechanisms of Diet and DNA Methylation?

Numerous studies have focused on the link between diet and DNA methylation in mammalians to elucidate the dietary exposures that may have lifelong consequences on epigenetic marks [12,49,50]. Different types of researchers (in vitro and in vivo) have presented the relationship between nutrition and DNA methylation, including prenatal and postnatal periods, showing that diets deficient in methyl donors and proteins may cause global DNA hypomethylation, or that high-fat diet consumption may result in changes in DNA methylation [1,51,52,53,54]. One of the most popular models that has studied the link between diet and DNA methylation is the ‘yellow agouti (A^vy^) mice’ model. The agouti gene is responsible for the regulation of brown/black (eumelanin) and yellow (pheomelanin) pigmentation in the mammalian coat. It has been shown that dietary methyl donors’ supplementation of dams can change the coat color by correlating with the A^vy^ methylation status [55]. However, the agouti mice model and most of these studies were incapable of showing the underlying epigenetic mechanisms regarding whether the DNA methylation occurred due to the expression or inhibition of special binding sites of methylation enzymes, substrates, cofactors, or something else. Besides, there are other questions that need to be clarified, especially regarding the nutrient doses and the duration of dietary exposure to DNA methylation [56]. 

Recently, evidence has suggested that nutrition affects the epigenetic regulation of DNA methylation in several possible epigenetic pathways: mainly, by altering the substrates and cofactors that are necessary for proper DNA methylation; additionally, by changing the activity of enzymes regulating the one-carbon cycle; and, lastly, by playing a role in several possible mechanisms related to DNA demethylation activity [1].

### 3.1. How Diet Influences Methylation Cycle and Methyl Donors

The key methyl donor for DNA and protein methyltransferases, SAM, is synthesized in the methionine cycle while accompanied by various nutrients present in the diet, including methionine, folate, choline, betaine, vitamins B2, B6, and B12 [57]. These nutrients act as precursors and contribute to the production of SAM, although they enter the cycle at different sites [1]. Therefore, any deficiencies in these nutrients may result in changes in the SAM pool, which can influence DNMTS’ reaction kinetics and DNA methylation, as well. Taking this opinion into account, many studies have started to investigate the link between SAM availability and its dietary sources, together with endogenous genetic factors [4].

Furthermore, after the removal of the methyl group, SAM is transformed into SAH, which is a strong competitive inhibitor of almost all methylation reactions, and also competes with SAM for the active site on the methyltransferase enzyme [58,59]. Since the supply of SAM and removal of SAH is necessary for DNMT activity, the SAM/SAH ratio has been suggested as a ‘methylation index’ to show the probability of DNA hypermethylation or hypomethylation [50]. Remarkably, some earlier studies have shown that SAH is an inhibitor of the DNMT-mediated DNA methylation [60,61]. Additionally, moderate elevations in plasma homocysteine concentrations have been shown to be associated with increased levels of SAH, but not SAM, and increased SAH levels have been associated with global DNA hypomethylation [59].

So far, the methyl and folate-deficient diets have been found to be largely associated with reduced levels of SAM, increased levels of SAH, and decreased SAM/SAH ratios in the livers of male rats and mice [62,63,64]. The changes in SAM and SAH levels also showed irreversible alteration in hepatic DNA methylation [63]. Moreover, a study showed that zinc deficiency has reduced the use of methyl groups from SAM in rat liver and resulted in global DNA hypomethylation [65]. A low-protein diet or undernutrition during gestation in mice and in utero in human studies resulted in both hypomethylation and hypermethylation at specific loci in offspring [66,67,68,69]. Although one study hypothesized that the hypomethylation of certain promoters upon protein restriction may be a consequence of decreased methyl group availability [66], most of the studies remained unclear regarding how diet changes the activity of DNA methylation, and they did not observe the upregulation of SAM, SAH, or DNMTs.

On the other side, high dietary methionine intake is believed to increase DNA methylation, and the methyl groups that are transferred in mammalian DNA methylation reactions are believed to eventually derive from methionine [55]. High doses of folate supplementation showed an increase in methylation and normalized gene expression at specific loci, which is believed to induce a substantial increase of the intracellular pool of the SAM and SAM/SAH ratio [70,71]. However, little is known regarding the effect of methionine or methyl donors’ supplementation, and the mechanisms of action on DNA methylation are not clear [50]. Very few studies have examined the epigenetic mechanisms of the effects of high methionine intake on DNA methylation. In an epigenetic mouse model, Tremolizzo et al. [72] studied the effect of methionine on SAM, SAH, methylation status, and the expression of the reelin gene in the frontal cortex. The study showed interesting results. For example, after 15 days of methionine treatment, brain SAH was found to be double, whereas SAM was not affected. The reduction in the SAM/SAH ratio would be expected to hypomethylate DNA, but it has been found that specific CpG sites in the reelin promoter were actually hypermethylated in the cortex of methionine-treated mice. Hence, the significant increase in CpG methylation appeared to downregulate reelin expression. A follow-up study from Dong et al. [73] showed that a 15-day methionine (MET) treatment increased the binding of methyl CpG binding protein 2 (MeCP2) to the reelin promoter, which is thought to be the factor behind hypermethylation. However, the same effect was not found in other control genes (*Gad65* and *β*-*globin)*. Another model examining MET-induced alterations in DNA methylation found no significant dietary effects on genome-wide DNA methylation, although methionine supplementation significantly decreased the SAM/SAH ratio in the liver and brain [74]. The problem with determining the SAM, SAH, and their ratio in order to examine nutritional influences on DNA methylation is complicated for several reasons. To start with, each mammalian cell is responsible for synthesizing its own SAM, and SAM cannot cross the plasma membrane. However, SAH does leak from the cell with excessive accumulation. Thus, interpreting the SAM/SAH ratio on a tissue-specific basis and the ratio in plasma may not provide a meaningful indication of systemic methylation [75].

Betaine is an important methyl donor, which can be produced by choline or taken through diet. Betaine converts into dimethylglycine (DMG) after it provides a methyl group to homocysteine [11]. Studies have suggested that plasma DMG is a good indicator of betaine utilization as a methyl donor [76,77]. Moreover, SAM can inhibit BHMT and reduce the usage of betaine as a methyl donor [10], and it is important for SAM to stimulate the BHMT pathway in order to sustain its concentrations [78]. Choline methyl-deficient diets showed reduced hepatic concentrations of SAM and increased levels of SAH in the livers of mice [62]. A rat study evaluating the choline-deficient diet for seven days also showed that the effects of choline deficiency on reducing liver methionine formation by 20–25%, SAM by 60%, and increasing liver SAH by 50% were significant [79]. Plasma SAM levels were found to be significantly correlated with plasma levels of choline and DMG, but not with betaine [80]. To date, evidence has also shown that folate deficiency may lower choline and betaine levels in liver, or that choline deficiency may decrease hepatic folate stores, and thus can affect the methyl transfer of one carbon cycle in the liver [81,82]. On the other hand, a study showed that folic acid-supplemented, BHMT-deleted mice have produced more hepatic SAM compared to BHMT-deleted mice fed a folate-deficient diet or a control diet [83]. It has been a long time since a diet very low in choline and methionine resulted in the decreased methylation of cytosine in the liver [84,85,86,87]. However, studies have failed to show the direct interaction between choline, biotin, and DNA methylation through SAM and SAH activities or different mechanisms, if available.

Ultimately, most of the studies did not show the biochemical mechanisms of how methyl donors lead to aberrant DNA methylation. They relied heavily on assumptions. It is not clear how reduced levels of SAM or increased levels of SAH were causing global hypomethylation. Is it because there were not enough methyl donors to bind DNMTs? Alternatively, perhaps SAH was inhibiting the entry of the DNA nucleotide cytosine into enzymes’ active sites. It is believed that there is not a simple correlation between methyl donors and DNA methylation. Hence, more studies are warranted to explain the underlying mechanisms in order to contribute to set patterns of DNA methylation in cells.

### 3.2. What Are the Diet-Related Cofactor and Enzyme Activities in One-Carbon Cycles?

Enzymes taking a role in the folate cycle (MTHFR, MTR, MS, SHMT, etc.) are regulated by micronutrients such as vitamins B2, B6, and B12. It is assumed that supplementing diets with these micronutrients may contribute to the maintenance of DNA methyl marks and therefore regulate DNA methylation [71]. Additionally, it is believed that variations in the bioavailability of these micromolecules may affect DNA methylation by altering the activity of the one-carbon cycle and the production of SAM [1].

MTHFR is an essential enzyme for the maintenance of the folate cycle and methylation of CpG islands [88]. SAM is a strong inhibitor of MTHFR, which also makes it the major regulator of folate-dependent homocysteine remethylation [89]. MTHFR activity may deteriorate due to an excess concentration of methionine and SAM or polymorphisms, or a low concentration of its cofactor vitamin B2, which decreases the synthesis of 5-methyltetrahydrofolate and thus the remethylation of homocysteine [90]. Conversely, when SAM concentrations are low and cofactor levels are high, the remethylation of homocysteine may be favored [89].

Moreover, a reduction of MTHFR activity increases the 5,10-methylenetetrahydrofolate levels while it drops the 5-methyltetrahydrofolate levels, which in return may favor the synthesis of deoxythymidine triphosphate (dTTP) over the methylation of CpG, and therefore alter DNA methylation [88,90]. Additional research has suggested that subjects who are homozygous for the polymorphism (*C677T*) in the *MTHFR* gene exhibited a significantly lower level of methylated DNA, but only under conditions of low folate status [91]. In tissue culture, a study has shown that folic acid, vitamin B2, and *MTHFR C677T* polymorphism affect genome instability, and that high B2 concentration may increase the activity of MTHFR, which may lead folate to provide methyl groups for the methionine synthesis enzyme instead of for thymidylate synthase [88]. Furthermore, it has been suggested that low vitamin B2 concentration in the presence of low folate may maximize the risk of genome hypomethylation [88]. However, this study did not measure DNA methylation directly. Instead, it measured several markers related to genome stability and linked it with methylation. Unfortunately, most of the evidence from in vivo studies has not clarified the direct link between folate cycle enzymes or cofactors and DNA methylation. More studies are warranted in order to evaluate the interaction between diet-enzyme activities in the one-carbon cycle and DNA methylation.

### 3.3. How Diet Affects the DNA Methyltransferase Activity

Li et al. were the first scientists showing the *DNMT1* gene leading to the genome-wide loss of DNA methylation and embryonic lethality in mice [92]. Numerous other studies later underlined the link between DNMTs and DNA methylation [93]. Besides, it is believed that those genetic modifications and the DNMT’s activity can be modified by nutritional factors. Animal studies reported that feeding methyl-deficient diets for nine weeks or longer caused DNA hypomethylation, which was associated with the suppressed expression of DNMT1 [94,95]. Lillycrop et al. showed a significant decrease of DNA methylation following a protein-restricted diet in pregnant rats, and indicated that altered DNMT1 expression may provide a mechanism for the induction of the hypomethylation of specific genes and individual CpG, although they did not show how such targeting may occur [96]. In this section, potential nutrient-based epigenetic mechanisms mostly involving the inhibition of DNMTs and altered DNA methylation have been evaluated.

The studies outlined in Table 1 suggest that several diet compounds may directly affect the expression of DNMT, or that methyl donors from the diet may indirectly modify DNMT activity by changing the intracellular concentration of SAM [97]. These assumptions have been demonstrated for several bioactive food components such as epigallocatechin-3-gallate (EGCG), genistein, caffeic acid, ascorbate, etc. [1]. A study found that each of the tea polyphenols (catechin, epicatechin, and EGCG) and bioflavonoids (quercetin, fisetin, and myricetin) inhibited SssI DNMT and DNMT1-mediated DNA methylation in a concentration-dependent manner. EGCG was found to be a more potent inhibitor that had direct inhibitory interaction with the DNMTs and the catalytic site of the human DNMT1. Additionally, when epicatechin was used as a model inhibitor, kinetic analyses indicated that this catechol-containing dietary polyphenol inhibited enzymatic DNA methylation (indirect) in vitro, largely by increasing the formation of SAH. [98]. Moreover, the treatment of the human esophageal KYSE 510 cell line with EGCG showed a dose and time-dependent reversal of hypermethylation and the re-expression of mRNA of *p16^INK4a^*, *RARβ*, *MGMT*, and *hMLH1* genes. Reactivation of some methylation-silenced genes by EGCG was also demonstrated in human colon cancer HT-29 cells, prostate cancer PC3 cells, and KYSE cells [99]. Both studies tried to explain the underlying mechanisms between EGCG and DNMT by using the structural model, molecular docking, and binding energy analysis. They revealed that EGCG shows competitive inhibition of DNMT1 by forming hydrogen bonds within the DNMT1 catalytic-binding region, thus blocking the entry of the DNA nucleotide cytosine into its active site, and inhibiting the methylation process [98,99]. Several other studies also revealed that EGCG decreased global DNA methylation levels, and also showed a protective effect by inhibiting the promoter hypermethylation of specific genes. These effects were attributed to the decreased mRNA and protein expression activity of DNMT1 and EGCG inducing the binding domain of DNMT1 to the promoter of the specific genes [100,101,102,103].

Genistein also showed a dose-dependent inhibitory effect on recombinant DNMT1 activity, and also decreased DNMT activity in nuclear extracts from KYSE cells, but this activity was found to be weaker than that of EGCG. However, six days of genistein treatment did not affect the mRNA expression levels of DNMTs and the methyl-CpG binding domain 2. Although genistein was found to have a synergistic or additive effect on DNMT inhibitors because it is a weak inhibitor of DNMTs, genomic global hypomethylation was not expected to occur after the dietary intake of soy isoflavones [104]. Another study showed that a genistein diet (300 mg of genistein/kg) was positively correlated with alterations in prostate DNA methylation at CpG islands of specific mouse genes. However, the mechanistic role of genistein was not examined [105].

Lee et al. revealed the effect of several other catechol polyphenols on DNMT activity. It has been shown that quercetin, fisetin, and myricetin may inhibit DNMT activity by transferring SAM to SAH [98]. The same group also showed that two common coffee polyphenols, caffeic acid and chlorogenic acid, have inhibited DNA methylation, which was catalyzed by prokaryotic CpG methylase (M.Sssl) DNMT and human DNMT1. The inhibition of DNA methylation by caffeic acid or chlorogenic acid was found to be concentration-dependent, and the inhibition was predominantly through a non-competitive mechanism, which suggested that it was due to the increased formation of SAH [106]. Eventually, caffeic acid/chlorogenic acid treatment in cultured human breast cancer cells showed no significant change in the global methylation status. However, the concentration-dependent inhibition of DNA methylation in the promoter region of the *RARβ* gene was detected, which showed a potential inhibition effect in the promoter region [106].

Curcumin, an antioxidant component of a spice called turmeric, has been investigated by some study groups for its effect on DNA methylation [107]. Liu et al. suggested that curcumin covalently blocks the catalytic thiolate of DNMT1 to exert its inhibitory effect on DNA methylation by using molecular docking [108]. Moreover, a combination of curcumin with the hypomethylating agents increased the response to the drug in breast cancer patients [109]. However, Medina-Franco et al. suggested that curcumin has no significant effect on DNMT inhibition and global hypomethylation after following a multistep docking approach [110]. Thus, more studies are required to detect an interaction between curcumin and DNA methylation.

Parthenolide, a component of a plant called feverfew, has been used for the treatment of several diseases. It has been suggested that parthenolide may have a potential role in inhibiting the activity of DNMT1 by blocking the enzyme’s catalytic site, and a study indicated that dose and cell type-dependent parthenolide treatment decreased DNMT1 protein levels and induced a decrease in global DNA methylation. The same study showed that parthenolide inhibited the DNMT1 analog M.SssI by blocking the functional thiolate of the enzyme. Although parthenolide’s binding energy is not as strong as EGCG, it has been suggested that it may be an effective DNA methylation inhibitor [111].

Mahanine is found in several Asian herbs and species, and it is an alkaloid from the leaves of the curry leaf tree (*Murraya koenigii*) and lime berry (*Micromelum minutum*). It is mostly studied for its anti-inflammatory and anti-mutagenic activity. [112,113]. Mahanine is thought to have an anti-proliferative activity, which was associated with the inhibition of DNMT activity, and hence, may prevent the hypermethylation of a specific gene in the prostate cancer cell line [114]. However, the mechanisms of action were not clarified.

Eventually, studies evaluating the consumption of polyphenols showed that in general, EGCG and several other polyphenols are promising candidates, especially for future cancer therapies, based on their influence on the epigenetic pathway. Most of these studies showed kinetics and possible mechanisms that alter DNA methylation. These include increasing SAH, inhibiting DNMT’s catalytic base, blocking the promoter sites of specific genes, or covalently binding to thiol groups of enzymes/transcriptional factors. However, future studies evaluating the underlying mechanisms are still needed in order to clarify the pathways of epigenetics.

### 3.4. Is There a Link between Diet and DNA Demethylation?

The reversal of DNA methylation is crucial, and abnormalities are often observed in anomalies and diseases. Genetic and modifiable factors such as diet may affect the regulation of DNA demethylation, and thus genetic regulations. However, DNA demethylation’s interaction with modifiable factors in mammals is still not well understood [37]. Recent epigenetic studies have tried to investigate the link between nutrition and active DNA demethylation, which is believed to lead to several modifications in DNA methylation. One study tried to clarify the DNA methylation status of the liver of mice fed the methionine–choline-deficient (MCD) diet (for a week) by measuring the amount of 5mC and investigating the involvement of the active DNA demethylation. The results showed that the expression of DNMT1 and DNMT3a was significantly increased on the MCD diet. In addition, mRNA expression of Tet2 and Tet3 was significantly upregulated on the MCD diet. However, no statistical differences for 5mC content and other demethylation enzymes were found [115]. It is believed that for better epigenetic investigations, long-term studies are necessary. The deletion of Tet2 was found to cause an extensive loss of 5hmC, which was accompanied by enhancer hypermethylation and delayed gene induction in the early steps of differentiation [116]. It is assumed that methyl-deficient diets that alter the expression Tet2 may contribute to hypermethylation in specific areas [115].

Some studies have shown that the presence of ascorbate (vitamin C) may modify the status of DNA methylation [117,118]. In embryonic stem cells, ascorbate caused the widespread DNA demethylation of nearly 2,000 genes [118]. However, it remains unknown whether the effect of ascorbate on DNA demethylation is due to an enhanced hydroxylation of 5mC. A study showed that ascorbate enhances 5hmC generation, most likely by acting as a cofactor for Tet methylcytosine dioxygenase to hydroxylate 5mC in mouse embryonic fibroblasts [117].

Pogribny et al. evaluated epigenetic changes during hepatocarcinogenesis, which was induced by diets deficient in methyl donors, in his review, and he commented that methyl donors’ deficiency sustains the demethylation of genomic DNA that occurs in methyl-deficient animal’s cytosine in their liver [87]. Further, the results of past studies have suggested that demethylation may be associated with decreased levels of SAM, increased levels of SAH, a decreased SAM/SAH ratio [86], and the changed activity of DNMTs [119]. However, the latest studies have demonstrated that DNA hypomethylation or demethylation induced by methyl-deficient diets might be attributed to the induction of uracil, 5hmC, and 8-oxodeoxyguanosine [95,120]. The presence of these products may significantly coordinate with DNMT1 and lead to the demethylation of DNA [121].

Less is known about the role of nutrition in the base excision repair system. In one of the few studies that has examined the five genes (*SMUG1*, *TDG*, *UNG*, *MBD4*, and *DUT*) that are involved in the repair system to identify polymorphisms and establish whether one-carbon nutrient status can further alter their effects, single nucleotide polymorphisms in *SMUG1*, *DUT*, and *UNG* genes showed an association with DNA uracil concentration. However, one-carbon nutrient status was not associated with DNA uracil concentration, and did not modify the effect of the single nucleotide polymorphisms [122]. An older study showed that folate deficiency impairs the DNA excision repair system in rat colonic mucosa [123], and folate status was found to be associated with uracil misincorporation and genomic instability in humans. However, both studies were not linked to DNA demethylation. Together, the evidence suggests that more studies are required in order to understand the demethylation pathways and the part that dietary factors play in demethylation. 

## 4. Conclusions

**Figure 3:**
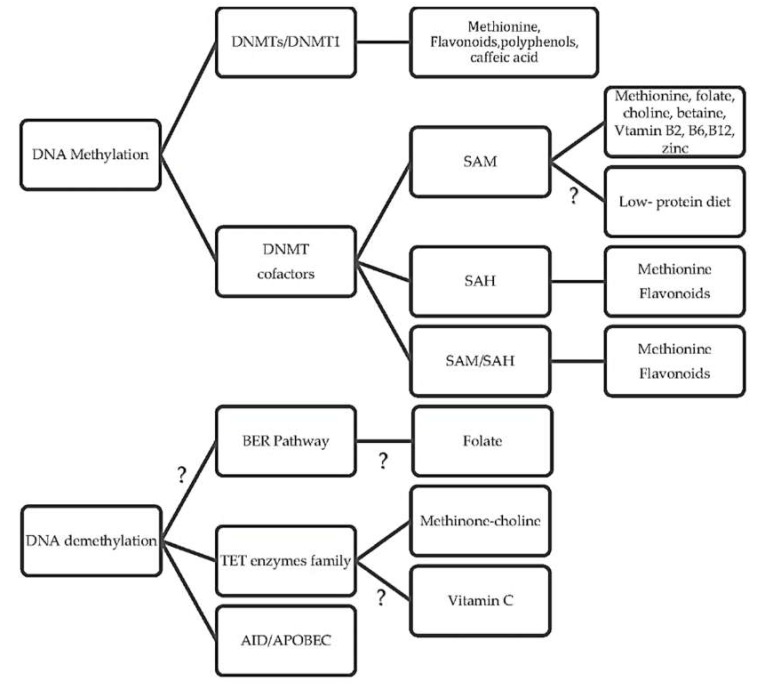
The possible mechanisms that nutrition can stimulate patterns of DNA methylation.

It is well known that nutrition has an indisputable influence on the epigenome. A great number of studies showed the changes in DNA methylation in specific genes, tissues, hormones, and cell lines after applying different diets [59,60,61,63,75]. These findings raise important questions about the diet-induced epigenetic pathways, such as: ‘What are the underlying regulatory pathways causing hypomethylation or hypermethylation?’ Recent evidence makes it clearer that the mechanisms regulating DNA methylation are very complicated, and that there is not one answer to this question. However, several possible assumptions were made for the interaction between diet and DNA methylation (Figure 3). It is suggested that nutrition may affect the epigenetic regulation of DNA methylation by altering the substrates and cofactors that are necessary for proper DNA methylation such as methyl donors, SAM, and SAH. These factors may impair the DNMT’s catalytic base, blocking the promoter sites of specific genes or covalently binding to the thiol groups of the enzymes. Likewise, nutrition-based cofactors may change the activity of enzymes regulating the one-carbon cycle and the production of SAM. Lastly, nutrition may have a role in several possible mechanisms related to DNA demethylation activity, which have been suggested to be a new epigenetic approach [11]. For example, changing the expression of Tet family enzymes by methyl–choline-deficient diets is believed to alter DNA methylation [123].

Diet influences organs, body systems, and epigenetics as well. It is extremely important for researchers to study the mechanisms of dietary implications on DNA methylation in order to determine the optimal concentration of macro nutrients and micronutrients for genome stability, which would provide a guide to establishing recommended dietary allowances for the prevention of genome damage and further diseases [124]. However, our knowledge of nutrition and epigenetic mechanisms are still limited. Future studies are required that should focus on the comprehensive understanding of nutrition-epigenetic mechanisms and biochemical pathways, especially those interacting with enzymatic functions rather than just showing general modifications.

## Figures and Tables

**Table 1:** Studies that have evaluated the interaction between bioactive dietary components ^1^ and DNMT’s activity ^2^.

Studies	Dietary Components	Enzymes Inhibited or Expressed	Epigenetic Outcomes
Lee, W. J., et al. [98]	EGCG	DNMT1	EGCG inhibited human DNMT1 activity by binding in the catalytic core region
Fang et al. [99]	EGC–EGCG	DNMT	EGC and EGCG showed competitive inhibition of DNMT1 and treatment of the KYSE 510 cell line. EGCG showed a dose and time-dependent reversal of hypermethylation and re-expression of mRNA of *p16^INK4a^*, *RARβ*, *MGMT*, and *hMLH1* genes
Nandakumar, V., et al. [101]	EGC–EGCG	DNMTs	EGCG reduced the activity of DNMTs by decreasing the mRNA levels and protein expression of DNMTs.
Zhang, B. K., et al. [100]	EGCG	DNMT1	EGCG inhibited the mRNA and protein expression activity of DNMT1 and downregulated binding to the promoter of DDAH2.
Shukla, S., et al. [103]	EGCG	DNMT	EGCG decreased the mRNA and protein expression activity of DNMT1, and increased the expression of unmethylation-specific GSTP1 promoter.
Pandey, M., et al. [102]	Green tea polyphenols, EGCG	DNMT1	A dose and time-dependent inhibition of DNMT activity and protein expression was observed.
Day et al. [105]	Genistein		Genistein diet was positively correlated with alterations in prostate DNA methylation at CpG islands of specific mouse genes.
Fang et al. [104]	Genistein	DNMT1	Genistein showed a dose-dependent inhibitory effect on recombinant DNMT1 activity, and also decreased DNMT activity in nuclear extracts from KYSE cells. However, no effect on the mRNA expression levels of DNMTs and methyl-CpG binding domain 2 was observed.
Lee and Zhu [106]	Caffeic acid, Chlorogenic acid	DNMT1, M.Sssl DNMT	The caffeic acid and chlorogenic acid inhibited the DNA methylation that was catalyzed by prokaryotic M.Sssl DNMT and human DNMT1, and increased levels of SAH.
Liu, Z., et al. [108]	Curcumin	DNMT1,	Curcumin covalently blocks the catalytic thiolate of DNMT1 to exert its inhibitory effect on DNA methylation.
Liu, Z., et al. [111]	Parthenolide	DNMT1, M.Sssl DNMT	Dose-dependent parthenolide treatment decreased DNMT1 protein levels and induced a decrease in global DNA methylation. The same study showed that parthenolide inhibited M.SssI by blocking the functional thiolate of the enzyme.
Minor, E.A., et al. [117]	Ascorbate (Vitamin C)	DNMTs, TET2-TET3	Ascorbate increased the expression of DNMT1, DNMT3a, and mRNA expression of Tet2 and Tet3.
Sheikh, K. D., et al. [114]	Mahanine	DNMT	Mahanine was associated with the inhibition of DNMT activity, and hence, prevented the hypermethylation of a specific gene in the prostate cancer cell line. However, mechanisms are not clarified.

^1^ EGCG, epigallocatechin-3-gallate; EGC, epigallocatechin; ^2^ DNMT, DNA methyltransferase; KYSE 510, oesophageal squamous cell carcinoma; p16^INK4a^, tumor suppressor protein; RARβ, retinoic acid receptor beta; MGMT, *O*-6-methylguanine-DNA methyltransferase; hMLH1, human mutL homolog 1; DDAH2, dimethylarginine dimethylaminohydrolase; GSTP1, glutathione S-transferase Pi 1; M.Sssl, CpG methylase; SAH, S-adenosylhomocysteine; TET, ten-eleven translocation.

## Abbreviations

MTHFR	Methylenetetrahydrofolate reductase
SHMT	Serine hydroxyl methyltransferase
MS	Methionine synthase
BHMT	Betaine homocysteine methyltransferase
DMG	Dimethylglycine
5mC	5-methylcytosine
5hmC	5-hydroxymethylcytosine
5hmU	5-hydroxymethyluracil
5fC	5-formylcytosine
5caC	5-carboxylcytosine
Thy	Thymine
DNMT	DNA methyltransferase
TET	Ten-eleven translocation
AID	Activation-induced deaminase
TDG	Thymine DNA glycosylase
SMUG1	Single-strand selective monofunctional uracil DNA glycosylase
SAM	*S*-adenosyl-l-methionine
SAH	*S*-adenosylhomocysteine
BER	Base excision repair
RFTS	Replication foci targeting sequence
BAH	Bromo-adjacent homology
UHRF1	Interacting protein E3 ubiquitin-protein ligase
NER	Nucleotide excision repair
dTTP	Deoxythymidine triphosphate
EGCG	Epigallocatechin-3-gallate
